# Fibromuscular dysplasia of mesenteric arteries: a rare cause of multiple bowel resections—a case report and literature review

**DOI:** 10.1186/s12876-021-01702-y

**Published:** 2021-03-22

**Authors:** Shuwen Du, Shanbing Yang, Kangmei Jia, Peng Du, Limin Zhang, Jiheng Wang

**Affiliations:** 1 Department of Gastroenterology, The Seventh Medical Center, Chinese PLA General Hospital, No.5, Nanmengcang Hutong, Beijing, China; 2 Department of Colorectal Surgery, Xinhua Hospital, Shanghai Jiaotong University School of Medicine, Shanghai, China

**Keywords:** Case report, Crohn’s disease, Behcet’s disease, Fibromuscular dysplasia, Bowel resections

## Abstract

**Background:**

Fibromuscular dysplasia (FMD) is a type of unexplained nonatherosclerotic vascular disease that usually involves the renal and internal carotid arteries and rarely involves the mesenteric artery. Mesenteric artery FMD is difficult to distinguish from Crohn’s disease (CD) and Behcet’s disease (BD) solely based on symptoms. Patients with mesenteric artery FMD can present with an acute abdomen, but case reports of patients who have a long medical history and undergo multiple bowel resections are extremely rare.

**Case presentation:**

The patient was a 45-year-old woman with an 11-year history of intermittent lower abdominal pain and fever. At the age of 34 years, she underwent right hemicolectomy and appendectomy due to an acute abdomen. She suffered from oral ulcers between 34 and 36 years old. A clinical diagnosis of presumed CD was made by the age of 41, and she was treated with mesalazine; however, the effect was poor. At the age of 42, she came to our centre, and based on her atypical symptoms and examination results, we thought she had CD. Hence, she was treated with glucocorticoids for 3 years. However, when she was 45, due to steroid dependence, thalidomide tablets were added. Unfortunately, she suffered from another episode of intestinal obstruction. Therefore, she underwent enterectomy. The postoperative histopathological diagnosis was mesenteric artery FMD. She no longer underwent pharmacotherapy after the surgery. Although she did not have any of her previous symptoms and postoperative colonoscopy showed no signs of recurrence, splenomegaly and abnormal routine blood results were still present.

**Conclusions:**

Patients with mesenteric artery FMD can present with an acute abdomen. In addition, the symptoms and endoscopic manifestations of mesenteric artery FMD may appear similar to CD and BD. Hence, it is difficult to make a clear clinical diagnosis and proceed with treatment. Mesenteric artery FMD often requires surgical pathology to confirm its diagnosis. For patients who suffer from this disorder, surgery may be the best choice to improve the patient’s quality of life.

**Supplementary Information:**

The online version contains supplementary material available at 10.1186/s12876-021-01702-y.

## Background

FMD is a type of unexplained nonatherosclerotic vascular disease that usually involves the renal and internal carotid arteries but rarely involves the mesenteric artery. Clinical symptoms of mesenteric FMD, including postprandial abdominal pain, incomplete intestinal obstruction, multiple or focal ulcers of the intestinal mucosa, abdominal distension, and abdominal vascular murmur, with or without fever, are difficult to distinguish from those of CD and BD. Notably, FMD of the mesenteric arteries can lead to multiple bowel resections. However, case reports of patients who have a long medical history and undergo repeated enterectomies are extremely rare. To date, there are very few studies and only limited literature reporting a few cases with this condition in Asia, Europe, North America and other regions.

## Case presentation

The patient was a 45-year-old woman with an 11-year history of intermittent lower abdominal pain and fever. At the age of 34, she developed intermittent lower abdominal pain with irregular fever, which was easily induced after a meal. After undergoing computed tomography (CT), X-ray, ultrasound, and routine blood examinations, right hemicolectomy and appendectomy were performed due to an acute abdomen. In the postoperative specimen, a huge ulcer (7 × 5 cm) was found at the junction of the ileum and colon. The histopathological examination of the biopsy specimen showed chronic inflammatory changes in the intestinal tissue.

The patient had a history of oral ulcers between 34 and 36 years old. At the age of 41, intermittent lower abdominal pain accompanied by fever recurred. Colonoscopy (Olympus Corporation, #CF-Q260-AI, Japan) was performed in a local hospital, revealing a large ulcer in the terminal ileum. The histopathological examination of the lesion showed the formation of inflammatory granulation tissue. Plain films of the abdomen revealed incomplete intestinal obstruction. Splenomegaly was found on an ultrasound examination. The clinicians at the local hospital considered that she was likely to suffer from CD, so she was treated with mesalazine (4 g daily). The effect, however, was poor. When she was 42 years old, she was referred to our centre for systemic diagnosis and treatment.

The positive signs on the physical examination at admission were anaemia, weight loss (body mass index: 15.63 kg/m^2^), and an enlarged spleen. She had a history of anaemia for more than 20 years and no history of hypertension.

Routine blood examination revealed that her leukocytes were low at 2.55 × 10^9^/L (nl within 4–10 × 10^9^/L) and her thrombocytes were reduced at 70 × 10^9^/L (nl within 100–300 × 10^9^/L); her haemoglobin (Hb) was low at 77.0 g/L (nl within 110–150 g/L); anti-small intestinal goblet antibody was positive; C-reactive protein (CRP) was elevated at 57.5 mg/L (nl < 8 mg/h); and here erythrocyte sedimentation rate (ESR) was elevated at 28 mm/h (nl < 15 mm/h).

**Fig. 1:**
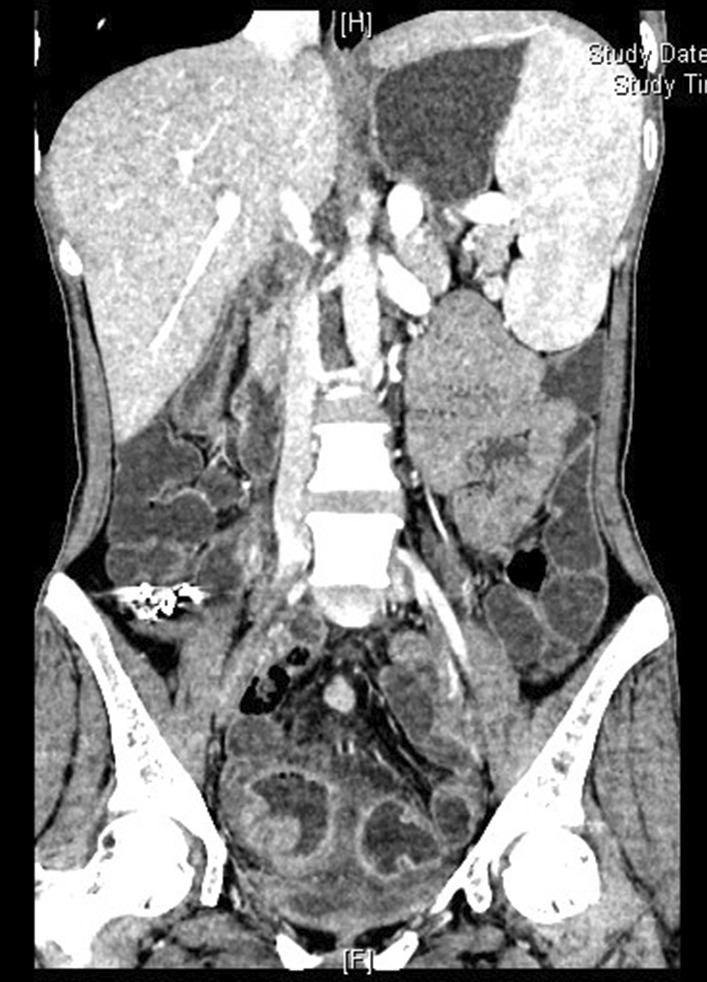
The small-intestine stimulation enhanced computer tomography (CT) examination revealed an unevenly thickened and strengthened wall of the sigmoid colon, splenomegaly, and portal hypertension

**Fig. 2:**
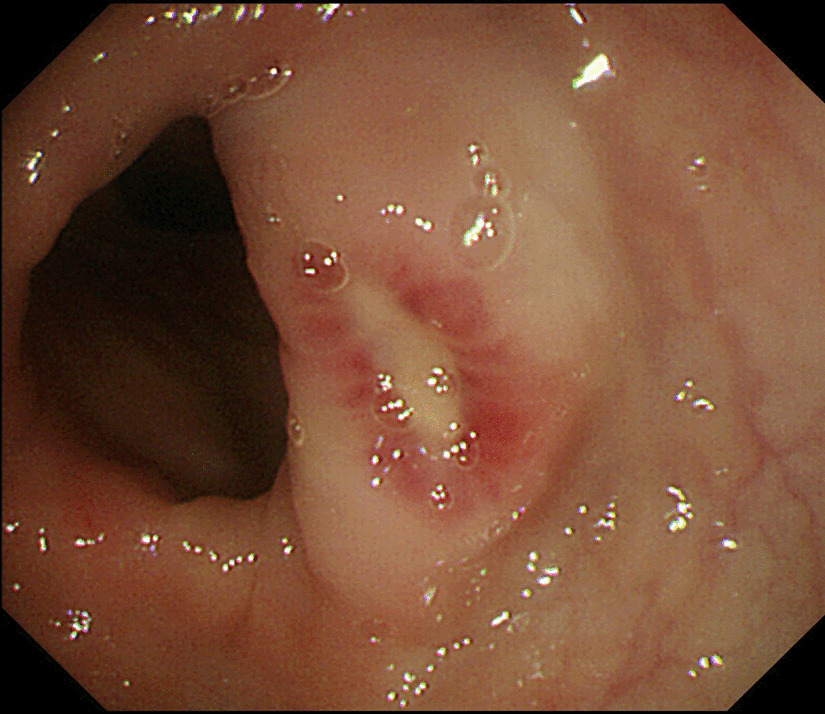
Colonoscopy revealed two ulcers in the small intestinal mucosa, multiple ulcers and erosions throughout the colon, and normal mucosa between the ulcers

The computed tomography enterography (CTE) examination revealed an unevenly thickened and enhanced wall of the sigmoid colon, splenomegaly, and portal hypertension (Fig. 1, Additional file 1: Figs. 1–6). Colonoscopy revealed two ulcers in the small intestinal mucosa, multiple ulcers and erosions throughout the colon, and normal mucosa between the ulcers (Fig. 2, Additional file 1: Figs. 7–9). Tuberculosis and haematological malignancies were excluded by performing related examinations.

Due to her atypical clinical symptoms and examination results, a diagnosis of CD was thought to be likely, although BD could not be excluded. She was treated with glucocorticoids for 3 years. Although her symptoms could be relieved in early treatment, intermittent lower abdominal pain accompanied by fever recurred after the dosage was reduced. Her symptoms could be relieved again after increasing the dosage. At the age of 45, due to steroid dependence, thalidomide tablets were added. Unfortunately, she suffered from another intestinal obstruction.

**Fig. 3:**
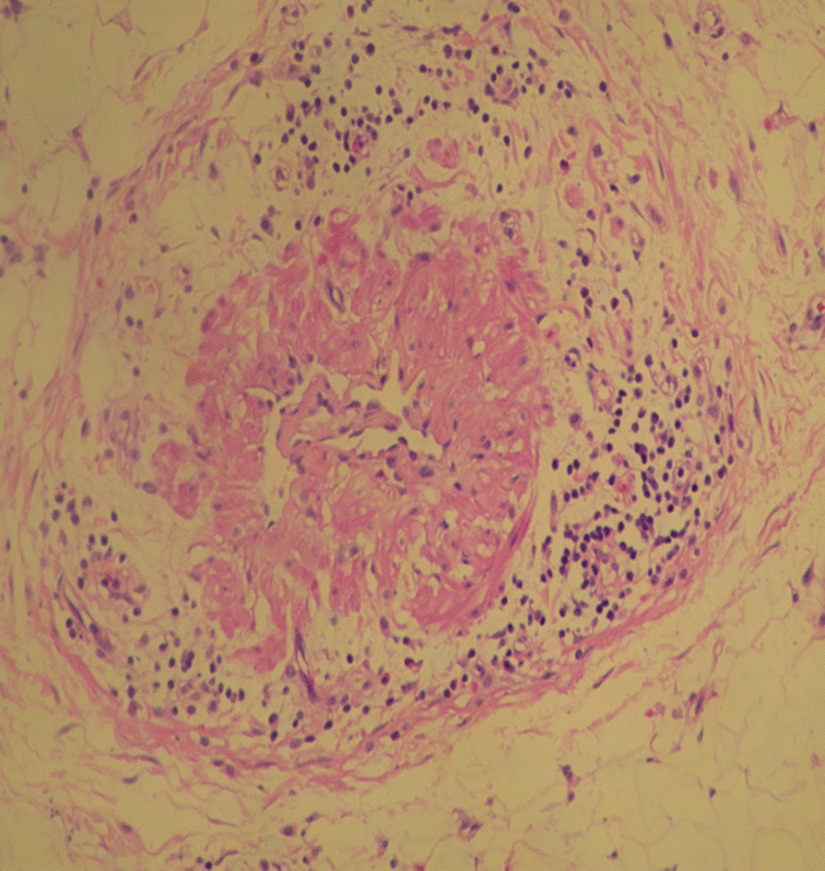
Postoperative histopathological examination showed inflammation of the arterioles of the intestinal wall, obvious stenosis or even occlusion of the arterial vascular lumen, proliferation of arterial smooth muscle and destruction of elastic fibres

**Fig. 4:**
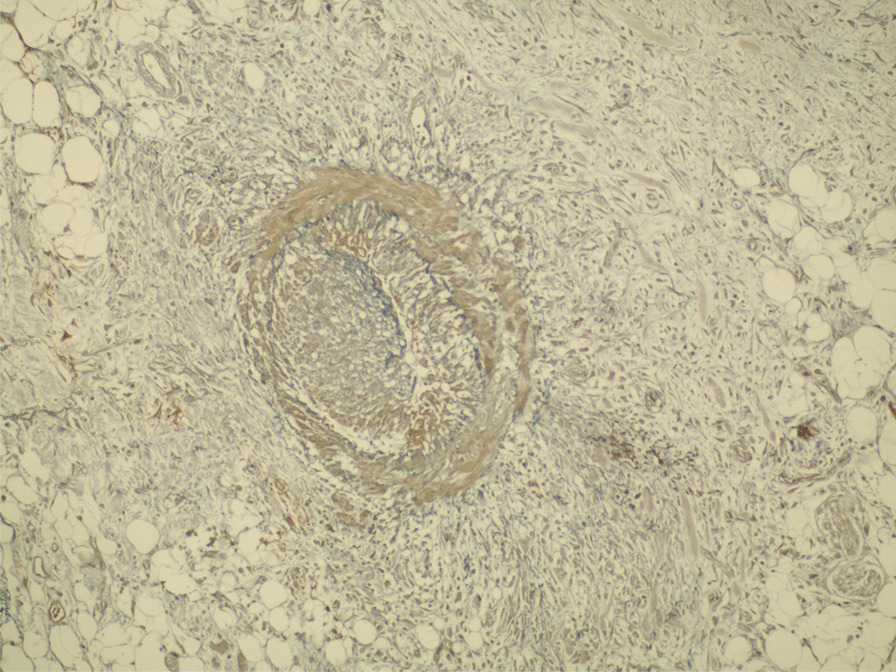
Postoperative histopathological examination showed inflammation of the arterioles of the intestinal wall, obvious stenosis or even occlusion of the arterial vascular lumen, proliferation of arterial smooth muscle and destruction of elastic fibres

Therefore, she underwent enterectomy of the diseased ileum (approximately 10 cm) and proximal ileostomy (Additional file 1: Figs. 10–11). Postoperative histopathological examination showed inflammation of the arterioles of the intestinal wall, obvious stenosis or even occlusion of the arterial vascular lumen, proliferation of arterial smooth muscle and destruction of the elastic fibres (Figs. 3 and 4, Additional file 1: Fig. 12).

The histopathological diagnosis was intestinal ischaemic injury caused by FMD of the mesenteric arteries (intimal fibroplasia). Her steroid medication was ceased after the surgery. In addition, antiplatelet therapy was not initiated. Although she did not have abdominal pain with fever again, and a re-examination by colonoscopy showed no signs of recurrence (Additional file 1: Figs. 13–14), splenomegaly (Additional file 1: Figs. 15–16) and her abnormal routine blood results still existed.

It is likely that her previous right hemicolectomy was also caused by ischaemic intestinal injury as a result of the FMD of the mesenteric arteries, and unfortunately the specimens from the previous right hemicolectomy were unavailable. Due to the obvious relief of her symptoms, she declined to undergo further CT examination of any other vessels and splenectomy (because she still suffered from cytopenia).

## Discussion and conclusions

FMD, or muscle fibre hyperplasia, is a non-inflammatory and non-atherosclerotic vascular disease that mainly involves the middle and small arteries [1]. FMD is characterized by a multifocal distribution (60–70 %) [2]. Young and middle-aged women are the most vulnerable [3]. Moreover, the renal and internal carotid arteries are the most commonly involved vessels; in addition, the vertebral artery, subclavian artery, axillary artery, mesenteric artery and splenic artery can also be involved [4]. The pathogenesis of FMD is still unknown, and smoking, homeostasis, mechanical factors, genetic factors and mural ischaemia may be related to FMD [4, 5]. Harrison and McCormack divided FMD into intimal, medial and adventitial types [6]. The medial type, the most prevalent, is characterized by multifocal thickening [2, 5]. The intimal type is likely to cause severe luminal narrowing, and the adventitial type shows circumferential thickening by fibroblasts and collagen [2]. As mentioned above, for patients with FMD, their vascular changes include stenosis, arterial dissection, and aneurysm, with or without lumen occlusion.

FMD can be clinically symptomatic or asymptomatic, and its haemodynamics can be variably affected, which mainly depends on the distribution of the involved vessels and the type and severity of the disease. FMD involving the mesenteric arteries is fairly rare [7, 8], and the clinical manifestations of the disease can be postprandial abdominal pain, mesenteric ischaemia, abdominal distension, abdominal vascular murmur, and multiple intestinal mucosa or focal ulcers. Furthermore, continuous or disordered adventitial and periadventitial smooth muscle proliferation can be seen in the histopathological examination of the involved mesenteric arteries and submucosa [7]. It is worth noting that patients with FMD of the mesenteric artery can present with an acute abdomen, but case reports of patients who have a long medical history and undergo multiple bowel resections are rare. Since our patient declined to undergo further examinations, we could not rule out the possibility of FMD involving the splenic artery, although FMD often involves multiple vessels.

In this case, FMD of the mesenteric arteries was mainly distributed in the patient’s intestinal wall (Additional file 1: Figs. 2, 6, 17). By reviewing the literature, it is interesting to note that FMD-like vascular changes can also be observed in postoperative specimens from patients with CD [1]. In contrast, mesenteric arteriovenous dysplasia/vasculopathy (MAVD/D) involves both arteries and veins. Mesenteric artery FMD is a disease lacking venous involvement. The clinical and histological features of these two diseases are similar to CD in some ways. Thus, it is difficult to make a precise decision throughout the clinical diagnostic and treatment processes.

For this patient, her clinicopathologic features were different from MAVD/D, which is usually diagnosed in older patients and involves small vessels (both arteries and veins) around the bowel wall that lack other multivessel involvement. In addition, the prognoses of these diseases are different. For patients with CD, pharmacotherapy can be used to control the disease, while surgery may be a curative method for patients who suffer from MAVD/D. In terms of patients with mesenteric artery FMD, which usually involves multiple vessels in other organs, surgery may be the best choice to improve the patients’ quality of life.

## Supplementary Information

**Additional file 1.** **Figure 1-6** The small-intestine stimulation enhanced computer tomography (CT) examination revealed an unevenly thickened and strengthened wall of the sigmoid colon, splenomegaly, and portal hypertension. **Figure 7-9**. Colonoscopy revealed two ulcers in the small intestinal mucosa, multiple ulcers and erosions throughout the colon, and normal mucosa between the ulcers. **Figure 10-11**. The specimen of the diseased ileum (approximately 10 cm) after the enterectomy.**Figure 12**. Postoperative histopathological examination showed inflammation of the arterioles of the intestinal wall, obvious stenosis or even occlusion of the arterial vascular lumen, proliferation of arterial smooth muscle and destruction of elastic fibres. **Figure 13-14**. Re-examination of the electronic colonoscopy after surgery showed no signs of recurrence. **Figure 15-16**. Re-examination of the CT after surgery showed splenomegaly still existed. **Figure 17**. The patients underwent abdomen CT revealed that her renal vessels were not involved.

## Data Availability

The data of this study are available from the corresponding author upon reasonable request.

## Abbreviations

CD: Crohn’s disease
Hb: Haemoglobin
ESR: Erythrocyte sedimentation rate
CRP: C-reactive protein
CT: Computed tomography
BD: Behcet’s disease
FMD: Fibromuscular dysplasia
