# Supplementary material for: Fibromuscular dysplasia of mesenteric arteries: a rare cause of multiple bowel resections—a case report and literature review
Source: BMC Gastroenterol. 2021 Mar 22;21:133. doi: 10.1186/s12876-021-01702-y (PMC7986496; doi:10.1186/s12876-021-01702-y)

Supplementary material

**Figure Legend**

**Figure 1-6.** The small-intestine stimulation enhanced computer tomography (CT) examination revealed an unevenly thickened and strengthened wall of the sigmoid colon, splenomegaly, and portal hypertension.

**Figure 7-9.** Colonoscopy revealed two ulcers in the small intestinal mucosa, multiple ulcers and erosions throughout the colon, and normal mucosa between the ulcers.

**Figure 10-11.** The specimen of the diseased ileum (approximately 10 cm) after the enterectomy.

**Figure 12.** Postoperative histopathological examination showed inflammation of the arterioles of the intestinal wall, obvious stenosis or even occlusion of the arterial vascular lumen, proliferation of arterial smooth muscle and destruction of elastic fibres.

**Figure 13-14.** Re-examination of the electronic colonoscopy after surgery showed no signs of recurrence.

**Figure 15-16.** Re-examination of the CT after surgery showed splenomegaly still existed.

**Figure 17.** The patients underwent abdomen CT revealed that her renal vessels were not involved.

**Figure 1**
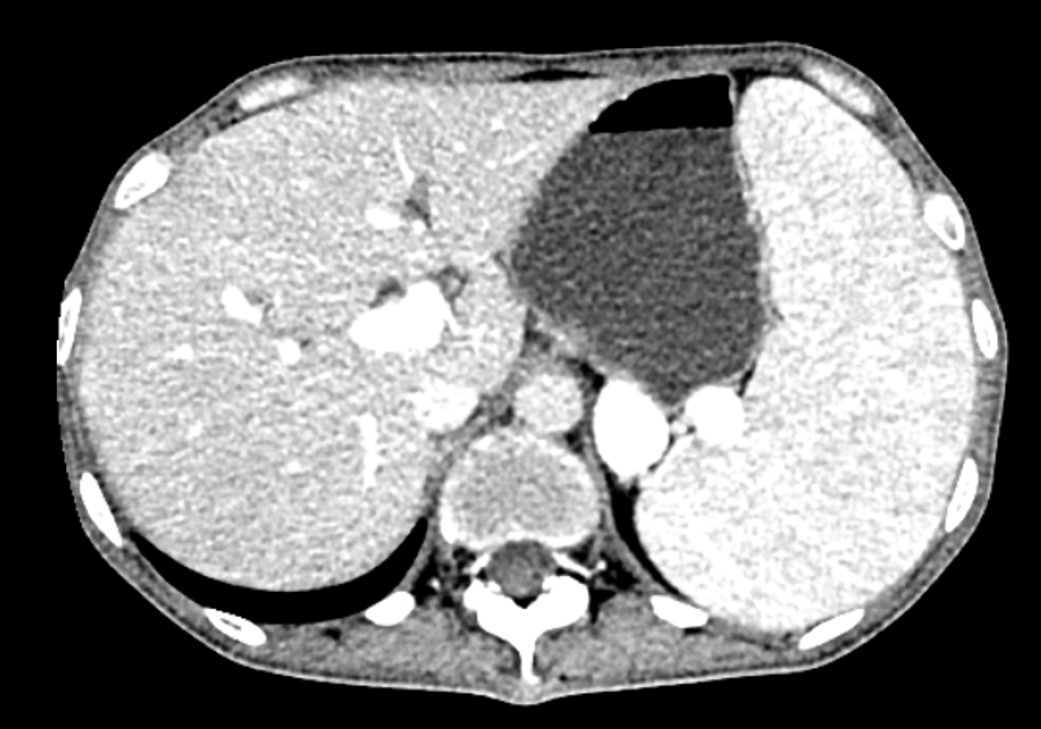


**Figure 2**

**
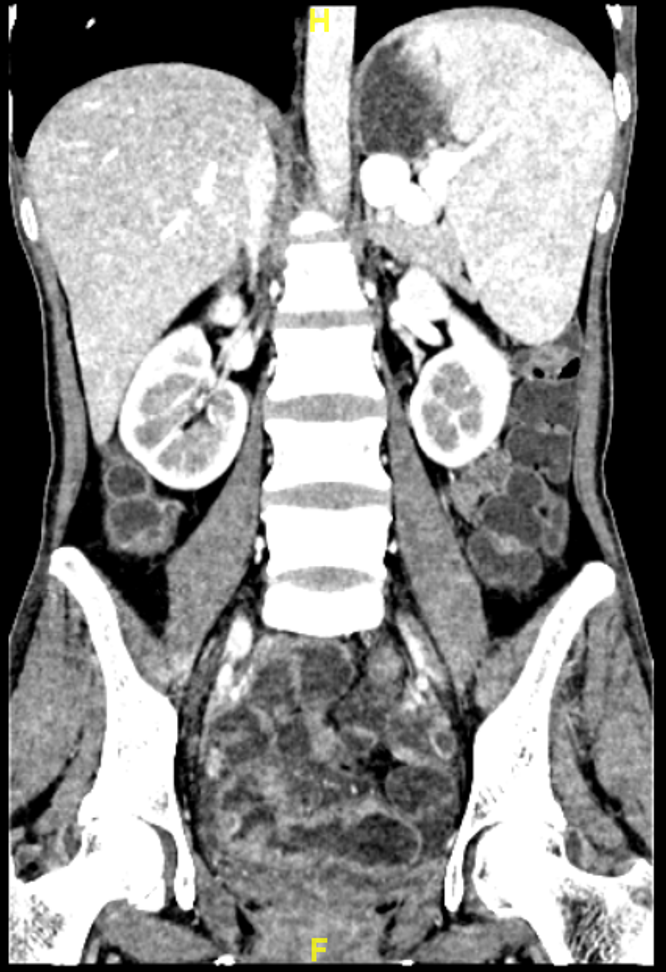
**

**Figure 3**


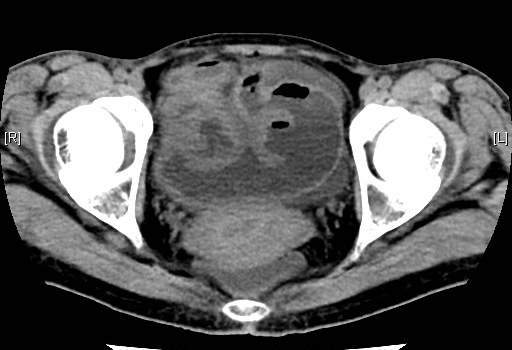


**Figure 4**


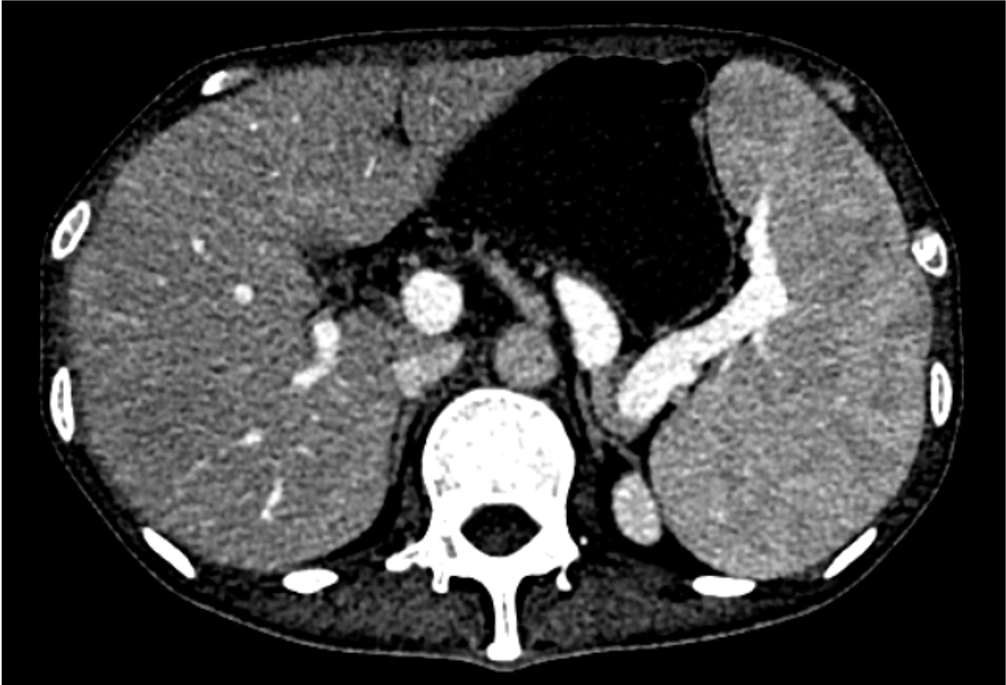


**Figure 5**


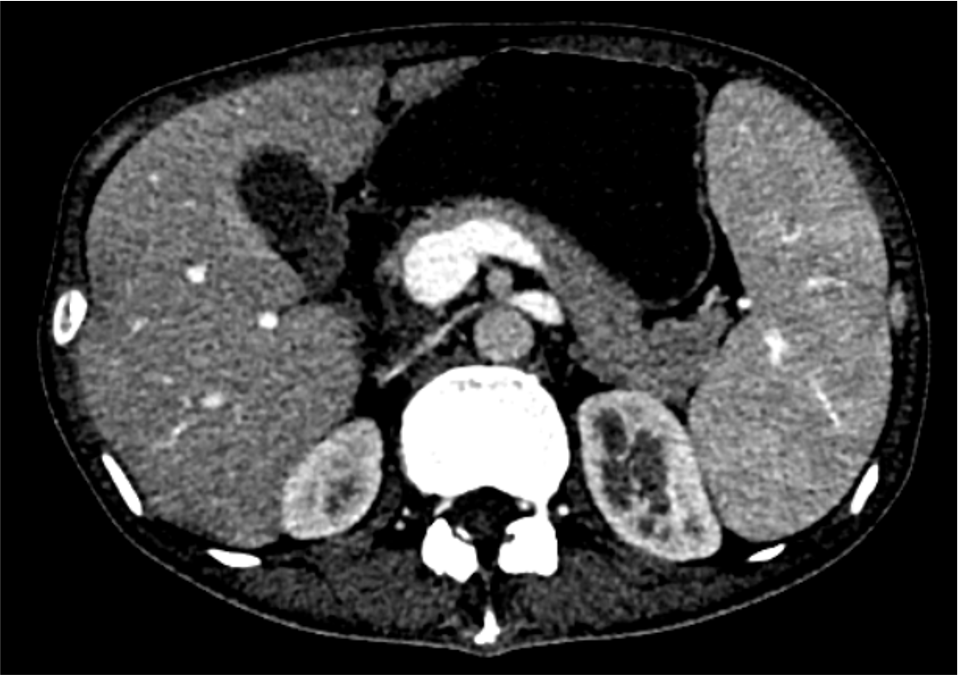


**Figure 6**


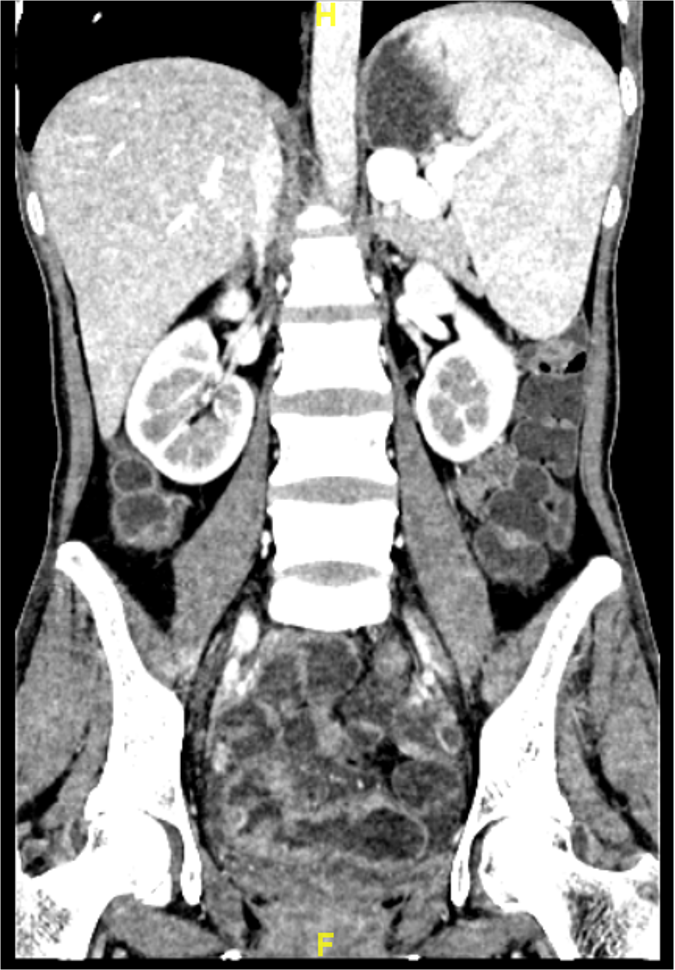


**Figure 7**


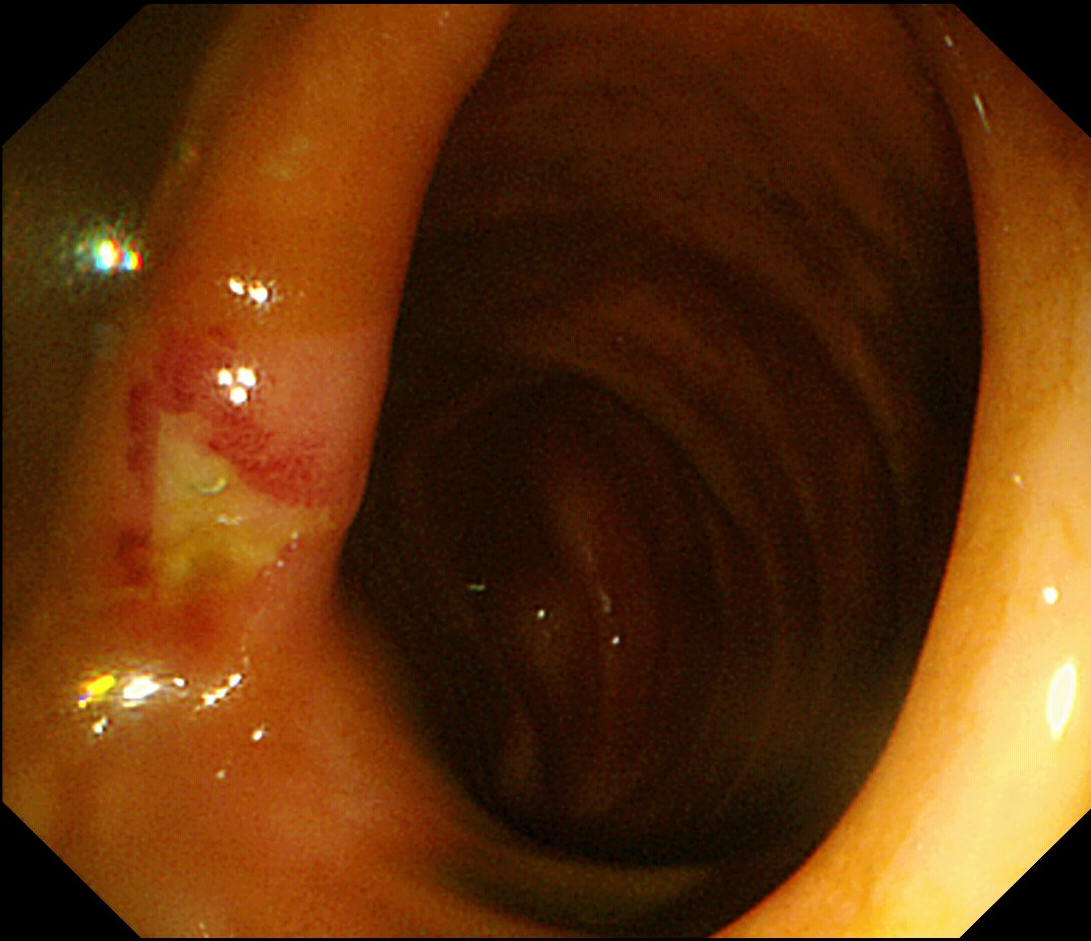


**Figure 8**


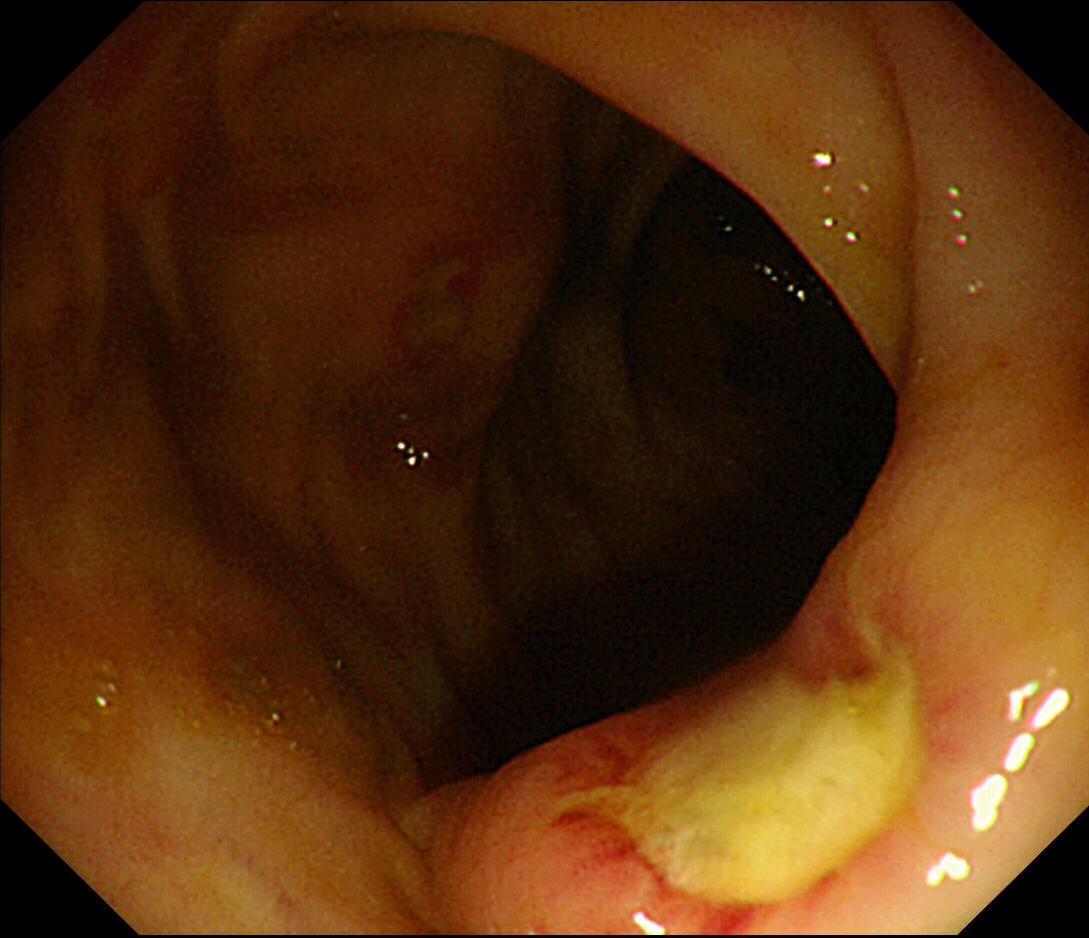


**Figure 9**


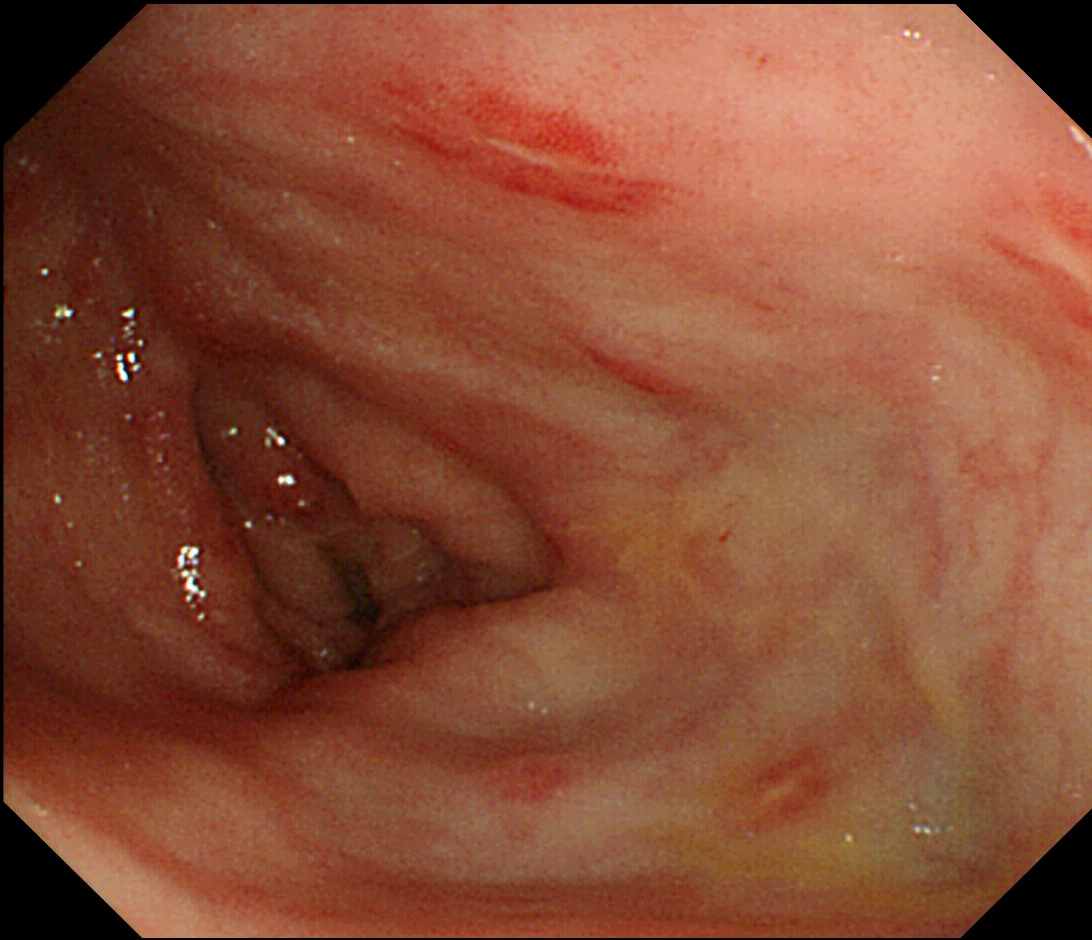


**Figure 10**


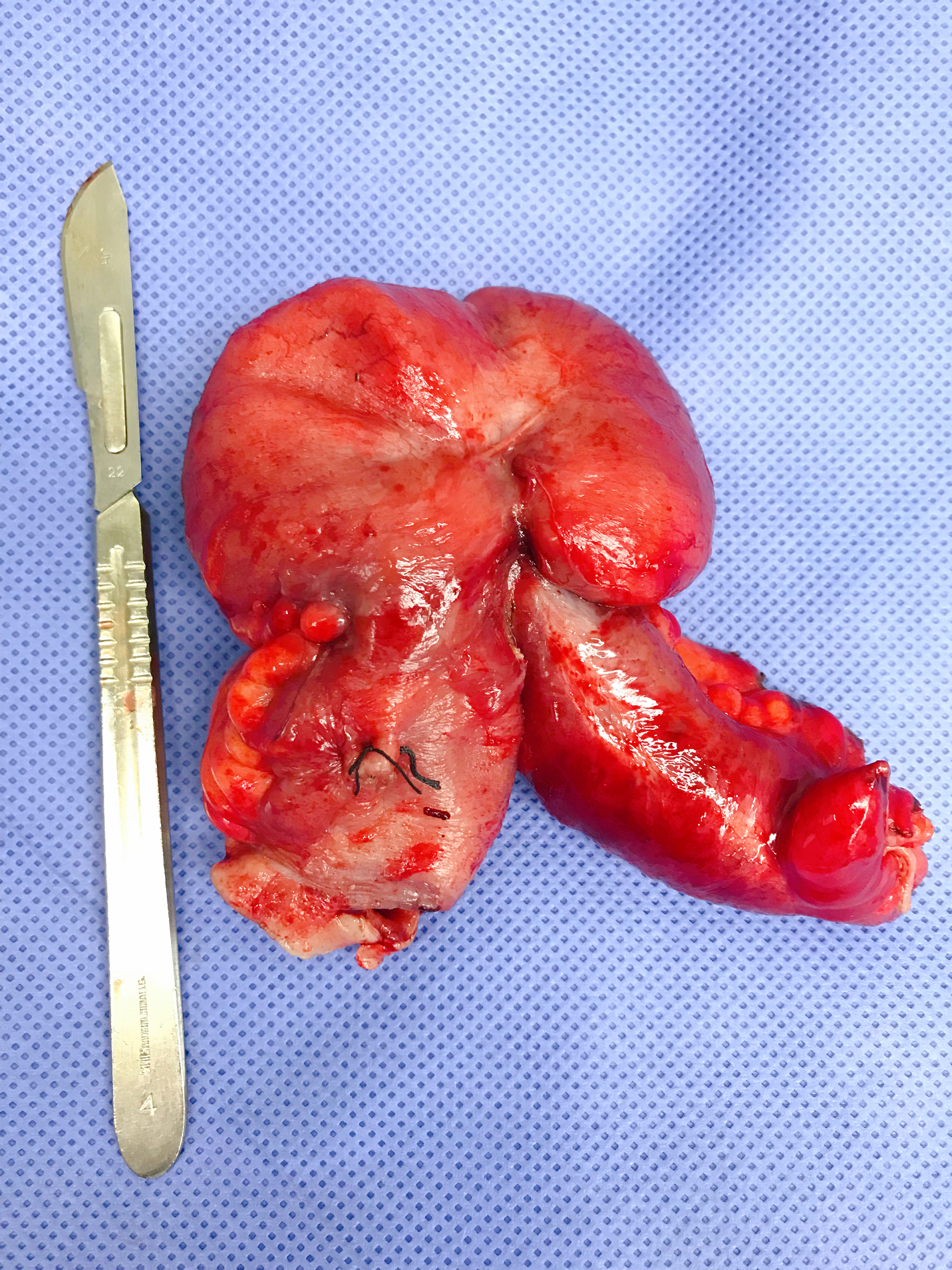


**Figure 11**


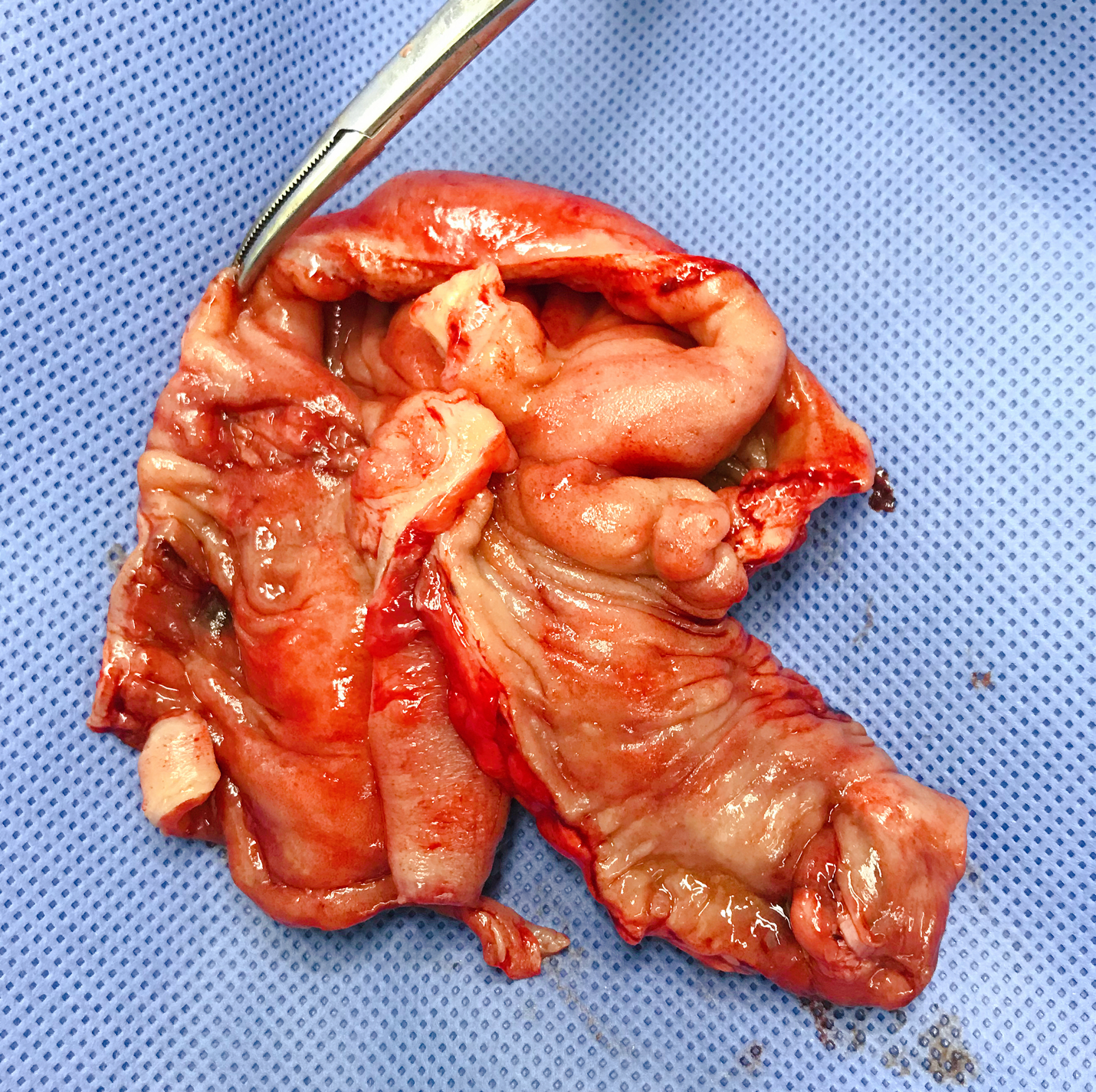


**Figure 12**


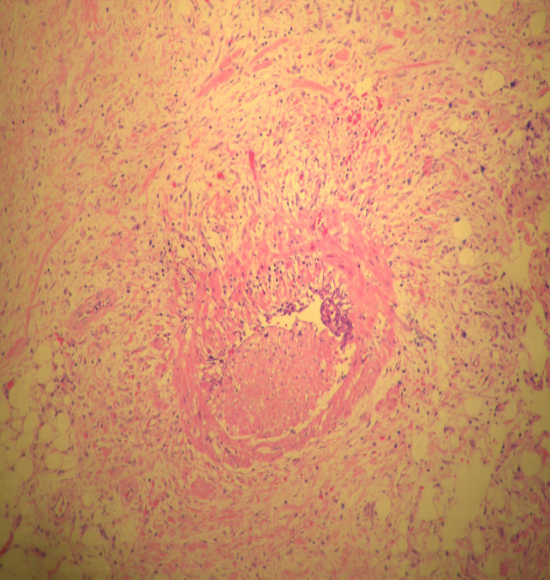


**Figure 13**


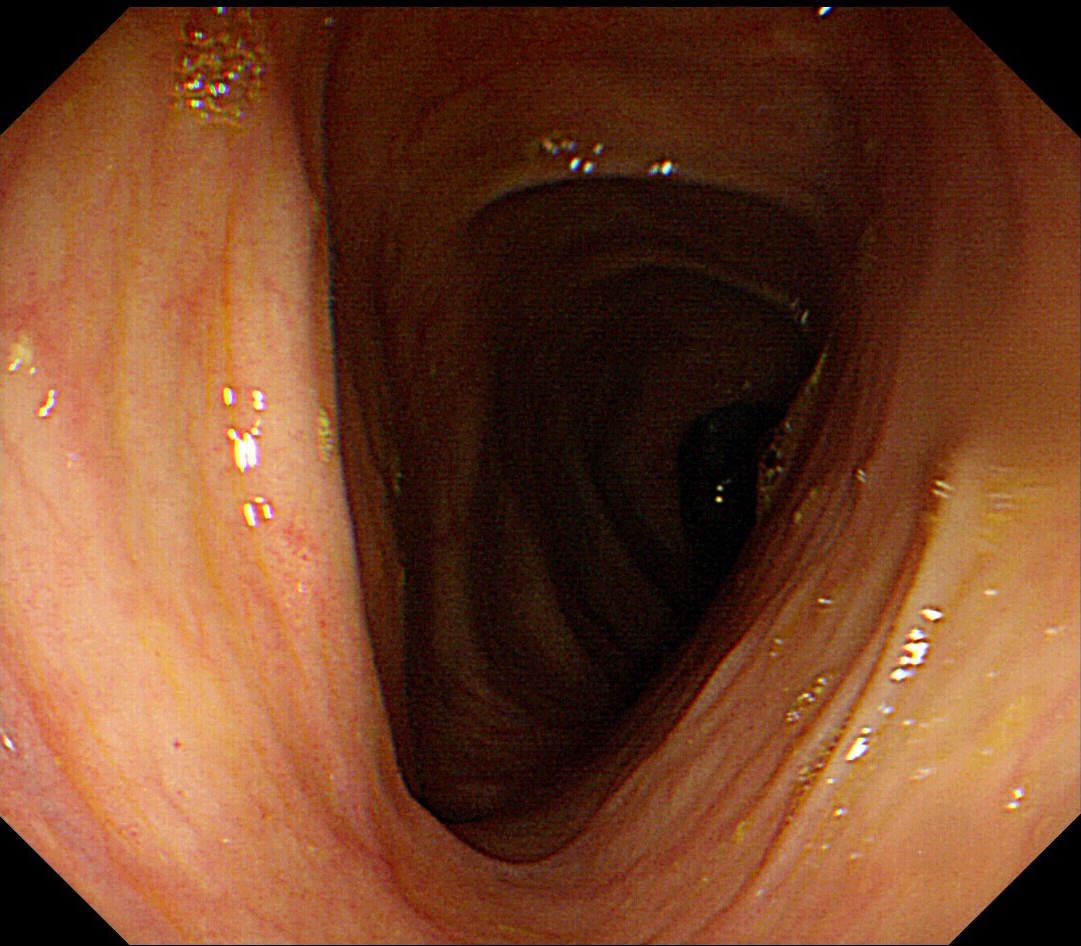


**Figure 14**


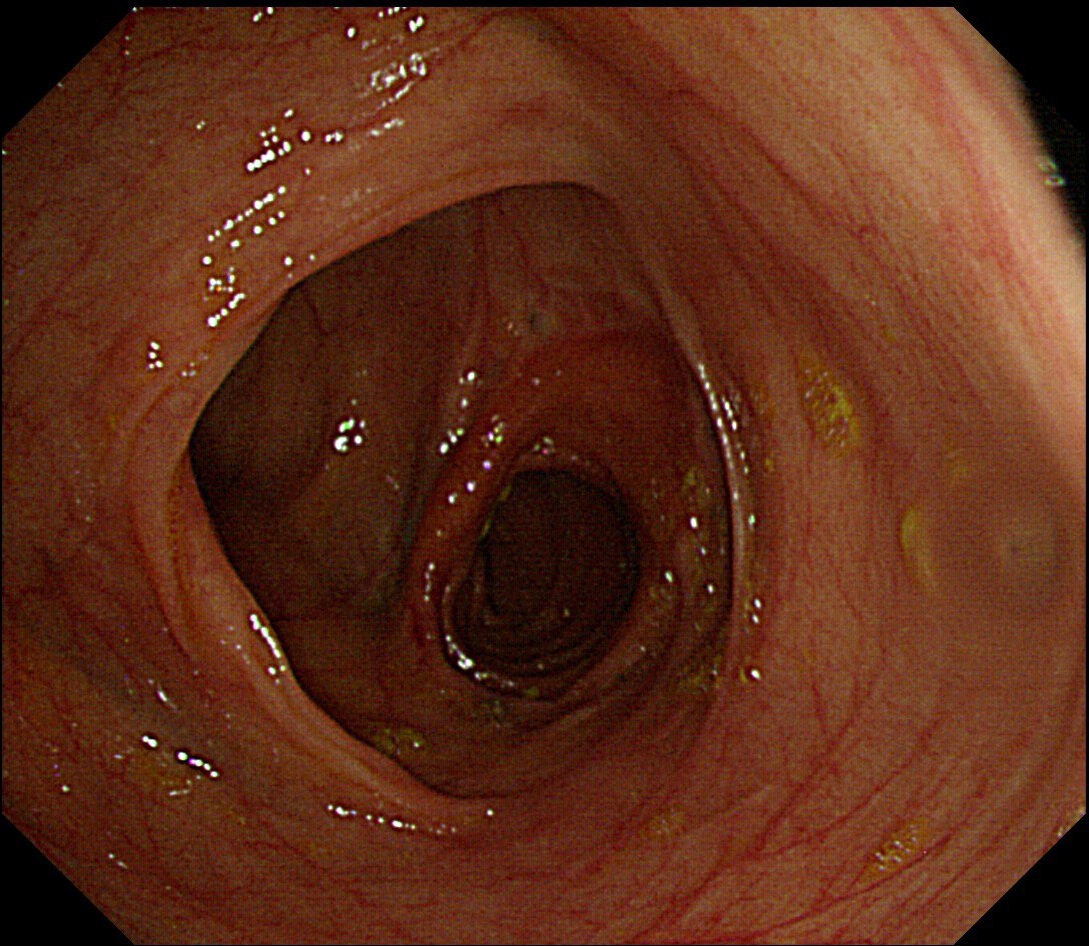


**Figure 15
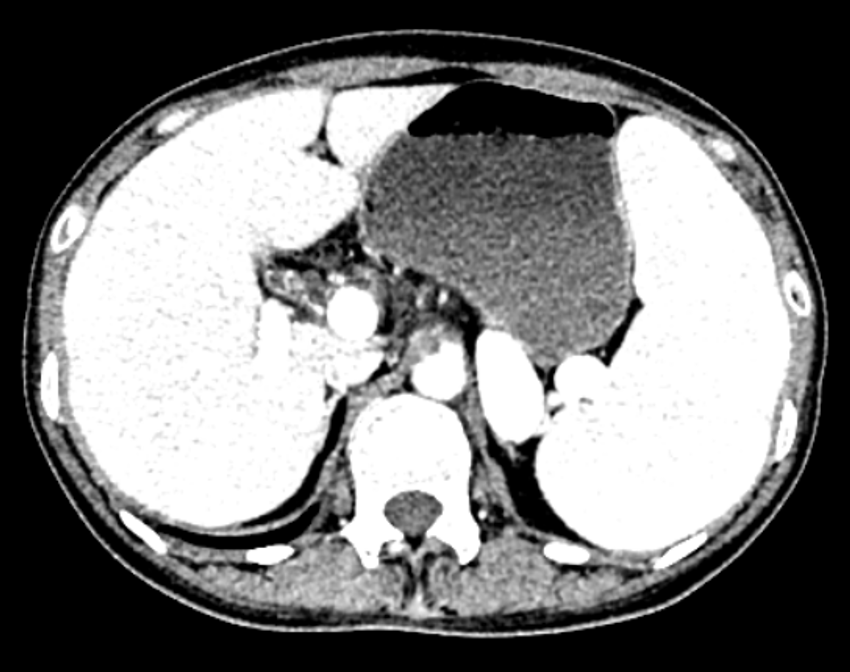
**

**Figure 16
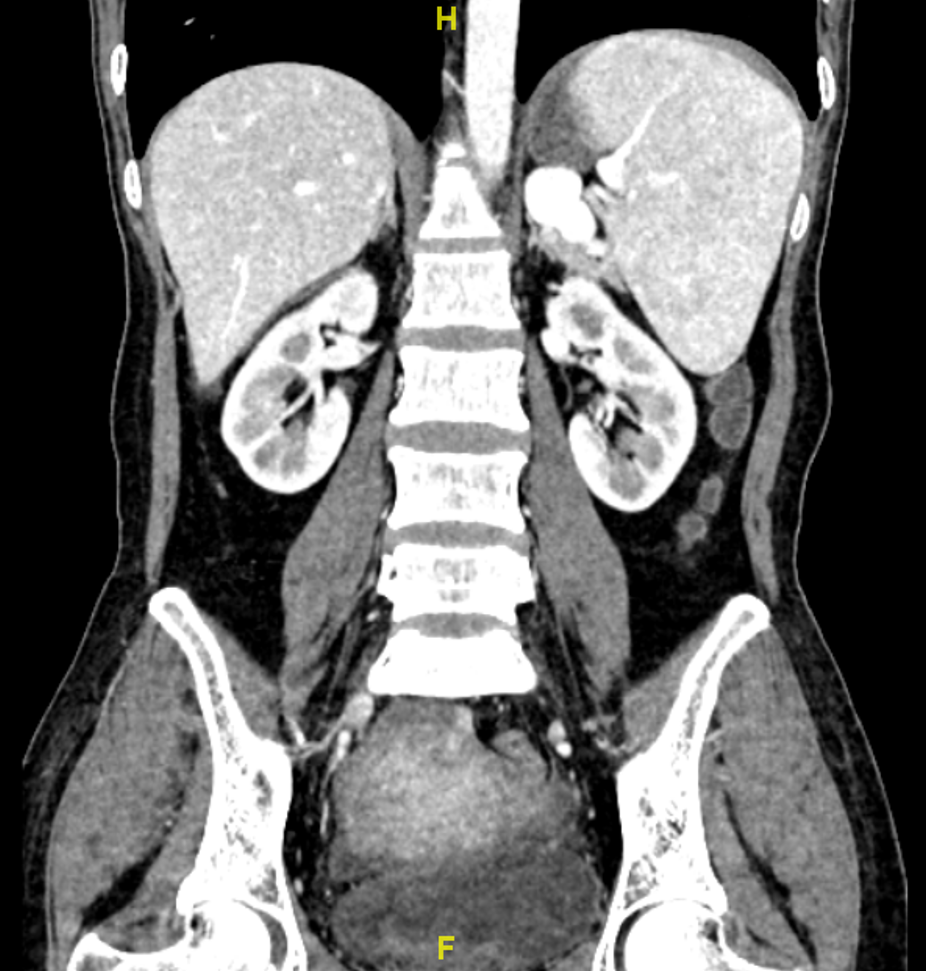
**

**Figure 17**


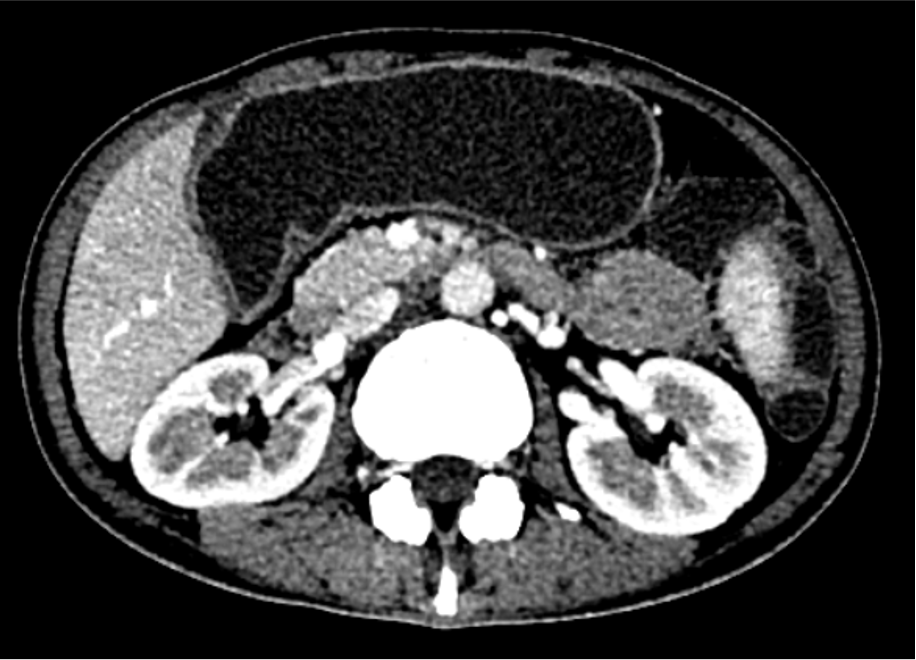

Supplement: Supplementary file 1 — Additional file 1. Figure 1-6 The small-intestine stimulation enhanced computer tomography (CT) examination revealed an unevenly thickened and strengthened wall of the sigmoid colon, splenomegaly, and portal hypertension. Figure 7-9. Colonoscopy revealed two ulcers in the small intestinal mucosa, multiple ulcers and erosions throughout the colon, and normal mucosa between the ulcers. Figure 10-11. The specimen of the diseased ileum (approximately 10 cm) after the enterectomy.Figure 12. Postoperative histopathological examination showed inflammation of the arterioles of the intestinal wall, obvious stenosis or even occlusion of the arterial vascular lumen, proliferation of arterial smooth muscle and destruction of elastic fibres. Figure 13-14. Re-examination of the electronic colonoscopy after surgery showed no signs of recurrence. Figure 15-16. Re-examination of the CT after surgery showed splenomegaly still existed. Figure 17. The patients underwent abdomen CT revealed that her renal vessels were not involved. [file 12876_2021_1702_MOESM1_ESM.docx]
